# CD40 stimulation as a molecular adjuvant for cancer vaccines and other immunotherapies

**DOI:** 10.1038/s41423-021-00734-4

**Published:** 2021-07-19

**Authors:** Timothy N. J. Bullock

**Affiliations:** Department of Pathology, University of Virginia, Charlottesville, VA USA

**Keywords:** CD40, vaccine, CD40L, T cell, dendritic cell, Tumour immunology, Immunization

## Abstract

The substantial advances attained by checkpoint blockade immunotherapies have driven an expansion in the approaches used to promote T cell access to the tumor microenvironment to provide targets for checkpoint immunotherapy. Inherent in any T cell response to a tumor antigen is the capacity of dendritic cells to initiate and support such responses. Here, the rationale and early immunobiology of CD40 as a master regulator of dendritic cell activation is reviewed, with further contextualization and appreciation for the role of CD40 stimulation not only in cancer vaccines but also in other contemporary immune-oncology approaches.

## Potential for cancer vaccines: days of future past

Undeniably, the treatment of cancer has been revolutionized by the introduction of novel immunotherapeutic agents that are administered as single agents or in combination with other cancer treatments. The clinical efficacy of checkpoint blockades and cellular therapies has prolonged lives in many cancer etiologies. However, it is also a hard reality that the majority of patients do not benefit from current immunotherapies, leaving us challenged to design and develop further iterations on successful therapies to expand their impact. In the case of solid tumors, the most sustained advances have been achieved with antibodies targeting checkpoint molecules that constrain immune cell function within the tumor microenvironment. However, the efficacy of these therapies, especially those targeting PD-1, generally correlates with the presence of T cells within the tumors. Thus, strategies to increase T cell presence, particularly cytotoxic CD8^+^ T cells, within tumors are an important focus. While cellular therapies have the potential to be immediately effective in this context, their activity in solid tumors has been modest. The basis for this observation is unclear, but some studies have suggested that immune cell trafficking and persistence within the tumor microenvironment (TME) is weak. These immune cells are potentially further compromised, particularly in the context of in vitro expanded cellular products with transgenic receptor targeting, by both their limited ability to form durable memory populations and the loss of the target antigen expression by the tumor. Thus, cancer vaccines, while initially promising in the tumor immunotherapy armamentarium, are now being strongly re-evaluated, as cancer vaccines can generate diversity in the immune response against target antigens and T cell differentiation states, both of which can promote trafficking and persistence at the tumor site. Further advances include the realization that a major source of antigens within tumors are inherent genetic aberrations and that the adjuvants used in combination with vaccines were initially underdeveloped. Here, we will focus on how targeting CD40, a TNF superfamily receptor expressed on a variety of immune cells, can be leveraged to improve cancer immunity in several vaccine-related settings, with the focus that most forms of vaccination will have limited therapeutic efficacy in the complex tumor microenvironment when the appropriate adjuvants are omitted from the vaccine regimen.

## Weaknesses of current adjuvants

The opportunity to develop vaccines in the context of cancer was initiated following the identification of immunogenic targets in tumors. However, most vaccine technology was initially based on the use of adjuvants (e.g., alum) that had been tested in the context of generating an antibody response. There are two traditional settings for the delivery of cancer vaccines: postsurgery, in a state of no radiographically evident disease but the likely presence of micrometastatic burden; and prevalent disease where metastatic spread has made surgery or radiation unsuitable for the patient. In the former, prophylactic vaccines are aimed at generating durable memory T cells that will reactivate upon re-exposure to the tumor antigen. In the latter, large numbers of effector T cells that can home to multiple tumor deposits are required, almost akin to cellular therapy. The vaccine strategies needed for these two scenarios may be quite different. Some studies have shown that the location of vaccine delivery impacts the homing ability of the responding T cells [1], while others have shown that elements of vaccine composition can also limit the systemic availability of responding T cells [2]. Thus, understanding and “vaccineering” both the quantitative and qualitative effects of different adjuvants is required to develop T cell responses that exhibit broad recognition of antigens and represent a spectrum of differentiation states.

Critical to the expansion of T cell responses to pathogens and tumors [3] is the activation of dendritic cells (DCs). Normally, DCs are present in peripheral tissues and acquire antigens via a variety of engulfment processes [4]. Upon sensing the presence of pathogen-associated molecular patterns (PAMPs) or damage-associated molecular patterns (DAMPs), DCs migrate to the lymph nodes and initiate encounters with naïve or memory T cell populations. On the basis of these interactions and a deepening of the understanding of innate pattern-recognition receptors such as Toll-like receptors (TLRs), NOD-like receptors, C-type receptors, RIG-1-like receptors and, more recently, the cGAS-STING pathway [5], innate sensors have begun to be exploited in cancer vaccines for their ability to promote the activation of antigen-presenting cells and the induction of T cell-supporting cytokines. However, few studies have consistently shown that targeting these innate sensors is sufficient to drive complete tumor control, perhaps because systemic induction of inflammation can perturb the chemokine gradients used by T cells to traffic to their target or that a high degree of toxicity occurs if they are delivered systemically. Thus, the alternative DC activation pathway of targeting CD40, the TNF superfamily at the nexus of innate and adaptive immunity, has the potential to serve this need.

## Molecular structure and signals of CD40

Seminal studies demonstrated that a critical step in the licensing of DCs to induce productive CD8^+^ T cell responses is the engagement of CD40 by CD40 ligand-expressing CD4^+^ T cells. This is the fundamental basis of CD4^+^ T cell-mediated “help”, without which CD8^+^ T cell responses are muted and memory is not properly formed. In the context of normal responses to pathogens, CD40 stimulation with CD40 ligand (CD154) is either provided by recently activated CD4^+^ T cells or, in some instances, by natural killer/T (NK/NKT) cells. This activation, or licensing, of DCs serves as a temporal bridge between CD4^+^ T cell activation and their indirect support of CD8^+^ T cell expansion [6–8]. However, in the context of cancer vaccination, these precursor populations are relatively rare, so developing agonists of CD40 that can serve as adjuvants for vaccines is a promising pathway to promote both CD4^+^ and CD8 T cell responses following vaccination.

CD40 is a 48 kDa type 1 transmembrane protein consisting of 193 amino acids. It is structurally divided into extracellular, transmembrane and intracellular domains. Its ligand, CD154, is a type II transmembrane protein with extensive posttranslational modifications, resulting in variations in molecular weight between 32 and 39 kDa. The extracellular structure of CD40L favors the characteristic trimerization of TNF superfamily members, which presents implications and complications in the design and development of agonistic molecules. Expression of CD40L is primarily found on activated T cells, although in some instances, it can be found on B cells and platelets and can be induced by inflammatory conditions on a variety of myeloid-derived cells [9].

**Fig. 1:**
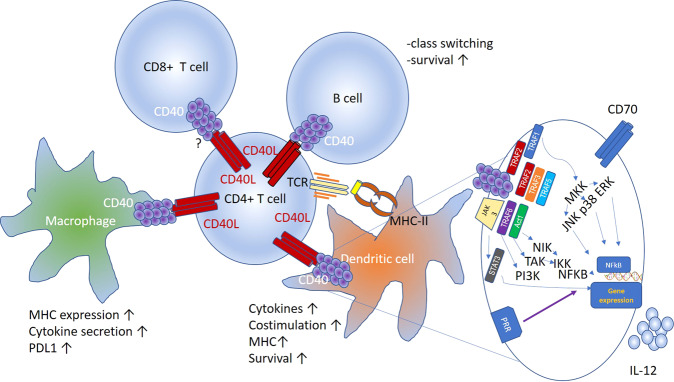
Schematic of the interactions between CD40L expressed by activated CD4^+^ T cells and other cellular components of the tumor microenvironment. The inset shows signaling pathways activated by CD40 stimulation in dendritic cells, leading to the expression of CD70 and IL-12

CD40 signaling primarily utilizes adapter proteins called TNF receptor-associated factors, resulting in the activation of both the canonical and noncanonical NFκB pathways, MAP kinase, PI3 kinase, and phospholipase-Cγ. Their activation leads to the characteristic downstream effects of these pathways, including transcriptional activation, cytoskeletal rearrangement and cell survival (Fig. 1). Other studies have indicated that CD40 can signal via JAK3-STAT5, and in the absence of this signaling, DCs induce T cell tolerance [10]. The extent to which these pathways contribute, individually or in combination, to the varied functional activities of DCs and DC differentiation has not been completely dissected. Furthermore, it is not yet known whether these pathways have different roles and outcomes in different cell types. Importantly, however, concerted and sustained CD40 signaling requires higher levels of oligomerization than those achieved through trimerization, which putatively supports more extensive engagement of the various signaling components. It is important to note that CD40 signaling pathways are quite distinct from those of pattern recognition receptors (PRRs), and while it is clear that the coactivation of these pathways has considerable functional consequences on DCs, how the two signaling pathways intersect has not been deeply studied.

## Functional consequences of CD40 stimulation

Focusing on DCs, CD40 engagement has a variety of functional outcomes, some of which are shared with PRR stimulation (Fig. 1). Both pathways increase the expression of antigen-presenting MHC molecules and the costimulatory molecules CD80 and CD86. The capacity of CD40 stimulation to activate DCs in combination with the fact that conventional DCs are highly specialized in their ability to cross-present antigens on MHC class I molecules leads to the conclusion that CD40-activated DCs are a lynch pin in the initiation of CD8^+^ T cell immunity. The TH1/TC1-promoting cytokine IL-12 is also induced by both CD40 and PRR stimulation. Given the toxicity that occurs with systemic delivery of IL-12 in clinical settings, CD40 stimulation provides a method for local delivery in a physiologically relevant context. Divergence is seen between these pathways with respect to two functionally important elements. CD40 stimulation, along with TRANCE-R [11], promotes the expression of antiapoptotic molecules, including Bcl-XL [12, 13], that promote DC survival, likely allowing a longer duration of antigen presentation in the draining lymph node to surveiling naïve T cells. Second, CD40 stimulation appears uniquely capable of inducing the expression of TNF superfamily members, including CD70, CD134 (OX40 ligand) and CD137 (41BB ligand). These “signal 3-like” molecules provide critical post-TCR/CD28 signaling that supports the continued expansion of effector T cells and, in particular, contributes to their differentiation and survival as memory T cells. Thus, there are multiple aspects of CD40 stimulation that naturally align with the goals of cancer vaccines, and when our lab prepares CD40-stimulated DCs for cellular vaccines in murine models, we perform quality control by assessing their expression of IL-12 and CD70. It should be recognized, however, that considerable synergy has been observed when CD40 and PRR stimulation occurs concomitantly [14–17], suggesting that vaccines that target both components will likely have increased efficacy.

While the ability of CD40 stimulation to support T cell responses is generally considered to be dependent upon its action on DCs, it is important to recognize that CD40 stimulation can target other cells within the tumor microenvironment and the tumor-draining lymph nodes (Fig. 1). In some cancer models, CD40-mediated activation of macrophages, which possibly mimics the effects of CD4^+^ T cell stimulation, has been shown to drive tumor control in an IFNγ-dependent manner, resulting in substantial remodeling and collapse of the tumor’s fibrotic network. It is interesting to consider this effect of CD40 stimulation on tumor fibrosis in the context of increasing the tumor’s accessibility to other therapeutics, including chemotherapies [18–20] and cellular products [21]. B cells also express CD40, and while their contribution of antibodies against tumor control remains controversial, there are data suggesting that CD40-mediated activation of B cells increases their antigen-presenting capabilities and modulates cytokine production, possibly supporting effector CD4^+^ T cell responses within tumors. Indeed, CD40-stimulated B cells have been explored as a cellular vaccine in several cancer models [22]. Finally, some murine studies have shown that CD40 can stimulate activated CD8^+^ T cells, providing a critical signal for their survival [23]. Whether human T cells receive such support via CD40 signaling has not been decisively demonstrated, although CD40 endodomains are being explored for CAR-T signaling [24].

## Preclinical integration of CD40 stimulation with tumor immunity

While a few studies have demonstrated the efficacy of CD40 stimulation as a monotherapy with agonists, these studies have generally occurred in the context of tumors that express strong antigens and have been shown to be generally responsive to many immunotherapeutic approaches [25–29].

More commonly, the ability of CD40 stimulation to control tumors has been performed with the addition of tumor-derived antigen. The earliest studies by Diehl et al. demonstrated that CD40 agonistic antibody stimulation could prevent the induction of tolerance to vaccines composed of peptides derived from shared tumor antigens [30]. Since this initial proof-of-principle study, numerous studies have shown that CD40-specific antibodies can provide a platform for peptide or whole protein-based cancer vaccines. Pertaining to the understanding that CD40 and PRR use discrete signaling molecules, seminal studies by Kedl and Seder showed that the addition of TLR agonists to proteins significantly boosted CD8^+^ T cell responses to protein vaccines, and directly conjugating TLR agonists was even more beneficial [31, 32]; this principle has been expanded to human studies with B cell-based vaccines [33]. Subsequent studies showed that expanded T cell responses were in part due to the elaboration of type 1 interferons (IFN-1) by activated DCs and the ability to target both the CD40 and PRR pathways to elicit CD70 expression on DCs [14]. Consequently, these data have led to the development of CD70-based agonists targeting CD27 as an alternative approach to targeting CD40 [34–37]. CD40-specific antibodies with TLR agonists, compared to either agent alone, significantly boosted the magnitude of CD8^+^ T cell responses to peptide-based vaccination in murine melanoma models [38–40]. Importantly, a recent study from our lab demonstrated that CD40 agonist vaccination with protein can elicit both CD4^+^ and CD8^+^ T cell responses to control murine melanoma with equivalent effectiveness [40]. Perhaps most striking, however, was the demonstration that blocking T cell egress from lymph nodes, the presumed site of CD40-mediated activation of DCs, did not impact either the expansion of intratumoral T cells or their ability to control tumor outgrowth. Thus, the tentative conclusion from this study is that CD40-based vaccinations may target intratumoral DCs and T cells. Given the recent studies that have demonstrated the critical importance of intratumoral DCs for tumor control [41], a logical next step will be to determine whether augmenting DC presence in tumors with DC mitogens such as FLT3 ligand [42] will further increase the activity of these vaccines. It also might suggest that some pre-existing T cell presence in tumors may be needed for CD40-based vaccines to be effective in the context of therapeutic cancer vaccines (as opposed to prophylactic vaccines).

While the efficacy of CD40-based cancer vaccines in preclinical models is clear, aside from the dependence on T cells, primarily but not absolutely CD8^+^ T cells, the mechanism of action of effective therapeutic CD40-based cancer vaccines is still under investigation. As mentioned above, CD40 stimulation mimics CD4^+^ T cell help and, as such, will convert tolerogenic protein vaccines to immunogenic vaccines [30]. In the previously mentioned study from our lab, within the tumor microenvironment, it was ascertained that CD40 stimulation promoted the activation of DCs by increasing CD86 expression and IL-12 secretion. At the level of T cells, several paradoxical changes in the frequency and function of T cells were observed. First, the massive increase in T cells seen with vaccination was primarily driven by a brief burst in the proliferation of phenotypically exhausted (PD1^+^IL2^‒^) T cells. Subsequently, the proliferative capacity and functional capability of these expanded T cells decreased, although the number of effector T cells remained higher than that of controls because of their vaccine-triggered expansion. Intriguingly, CD40 stimulation reduced PD1 expression on T cells but also decreased their expression of TCF-1, which has been associated with pluripotent T cells within the tumor microenvironment [43]. This may infer that CD40 stimulation drives further differentiation of T cells within the TME. However, a second round of vaccination to tumor-bearing mice with anti-CD40, polyIC and protein in the context of FTY720, which prevents T cells from leaving lymph nodes, resulted in additional tumor control, indicating that these intratumoral effector T cells were capable of a secondary response to vaccination. Going forward, understanding the relative contribution of pre-existing T cells compared to those that traffic from secondary lymphoid tissues will be important, as will whether immunization will synergize with checkpoint blockade therapies. Furthermore, understanding why the addition of an antigen promotes tumor immunity compared to adjuvants alone is worth understanding.

A criticism of cancer vaccines as an approach for tumor immunotherapy is that the tumor antigens that have been targeted to date are often (in the case of tumor-associated antigens, e.g., the melanocyte differentiation antigen gp100 or mesothelin) but not always (in the case of cancer-testes antigens, e.g., NY-ESO) derived from proteins expressed in the periphery by healthy cells. This could be expected to have induced a degree of self-tolerance, muting subsequent T cell responses to vaccination. The advent of genome sequencing aligned with algorithms that predict binding to MHC molecules has led to the development of next-generation cancer vaccines built on so-called “neoantigens” [44–47]. It is currently unclear whether neoantigen-based vaccines will need CD40 stimulation to overcome self-tolerance, but the cost-benefit between the toxicity of CD40 stimulation and the magnitude of CD40-driven T cell responses will need to be tested empirically in clinical trials.

Expanding on the theme that CD40 stimulation and PRR stimulation can be additive or synergistic but could be limited by cytokine-mediated toxicity, intratumoral delivery of a TLR7 agonist along with systemic CD40 stimulation has been shown to be effective in a murine model of mesothelioma [48, 49]. Furthermore, intratumoral TLR4 and CD40 stimulation, when combined with anti-PD1, has resulted in systemic rejection in a plethora of murine models [50]. In our recent study, a remarkably digital “responder” vs “nonresponder” phenotype was observed when melanoma-bearing mice were treated with anti-CD40 and polyIC without further tumor antigen treatment [40]. One possibility is that a threshold of antigens within targeted DCs needs to be met to support tumor-infiltrating DCs. Alternatively, differences in recruitment of immunosuppressive populations could account for variations in expanding T cell responses.

Analogous to protein-based vaccinations, CD40 stimulation has been deployed with DC-based vaccination [51]. Some initial efforts involved preactivating antigen-bearing DCs with CD40 stimulation to augment cytokine and costimulatory molecule expression [52, 53]. However, while these DCs have clearly ramped up immunogenicity, activated DCs often fail to traffic effectively to lymph nodes, instead staying at the injection site [54]. This can potentially be sidestepped by injecting the activated DCs directly into tumor-draining lymph nodes. Alternatively, CD40 stimulation can be provided after DC vaccination, allowing the activation of DCs once they have migrated to lymphoid tissues [55]. As mentioned above, promoting DC trafficking to tumors may also be a sensible strategy to consider prior to CD40 stimulation.

## CD40 stimulation beyond extrinsic vaccinations

The recognition that CD40 is a potent activator of DCs has led to studies that interrogate its use in autovaccination settings. Autovaccination refers to the process of an antigen being introduced to the host from an external intervention, such as the induction of tumor cell death by chemotherapy or radiotherapy. In an extensive series of studies, Vonderheide and colleagues first demonstrated that CD40 stimulation in combination with gemcitabine induced T cell-independent remodeling of the tumor stroma in humans, in particular inducing tumor-infiltrating macrophages to become tumoricidal and deplete the tumor stroma, which is important in pancreatic cancer [18–20]. Similar tumor microenvironment remodeling as a function of CD40 stimulation has been reported by other groups in different models [56, 57], indicating a common theme that above and beyond DC priming, CD40 can promote a proimmunity landscape within tumors. Whether consistent mechanisms, such as IFNγ production or metalloproteinase elaboration from activated myeloid cells, are critical for this, or whether CD40 stimulation shuts off the profibrotic activity of macrophages, remains to be elucidated. Subsequent studies in murine models showed the ability of CD40 stimulation to achieve TME remodeling as a monotherapy, and increased tumor control was achieved when CD40 stimulation and anti-CTLA-4 and anti-PD1 were used in combination with chemotherapy. In this instance, T cell responses were strongly induced, and curative protection that resulted in immunological memory and resistance to subsequent tumor rechallenge was achieved [20]. Further investigations indicated that CD40 stimulation synergized with radiotherapy and checkpoint blockade, demonstrating the elusive abscopal effect of controlling unirradiated tumors, which is indicative of a substantial systemic immune response. These data show a potentially encouraging role for CD40 in remodeling distal, untreated tumors, making them more permissive for T cell trafficking and infiltration. Anti-CD40 has also shown activity in the novel approach of using focused ultrasound to introduce nonionizing damage to tumors [58].

From these studies that show the benefit of adding CD40 stimulation to standard of care therapies or viral delivery, it could be inferred that the degree of DAMP or PAMP release from either infected or dying cells achieved by the primary intervention is perhaps insufficient to fully activate T cells. Alternatively, these conventional approaches are not sufficiently targeting CD4 T cells or NK cells that would normally provide CD40 stimulation to DCs.

While it is clear that DCs provide a critical link between innate and adaptive immune responses within the tumor microenvironment and draining lymph nodes [59–61], it is worth noting that, as with other myeloid cells within the tumor microenvironment, activated conventional DCs express PDL1 and PDL2, which serve as ligands for the PD1 checkpoint molecule [62]. The expression of high levels of PD1 on T cells is associated with dampened activity (reviewed extensively elsewhere). Host expression of PDL1 and PDL2 can significantly contribute to limitations in the T cell responses to tumor antigens [63, 64]. Some studies have indicated that CD40 stimulation can augment PDL1 expression on DCs and macrophages [65, 66], which may explain the need for anti-PD1 in the aforementioned chemotherapy and radiotherapy studies with anti-CD40. The addition of TLR stimulation in combination with CD40 stimulation can further increase PDL1 expression on DCs [67]. Not surprisingly, CD40-based immunotherapies have benefitted from the inclusion of checkpoint inhibitor blockade, commonly in the form of anti-PD1 [68–70]. It should be noted that there is a relative dearth in knowledge as to whether PDL1 and other checkpoint molecules are similarly regulated on cDC1 and cDC2 by CD40 stimulation and whether the location of these DCs within tumors or lymph nodes influences the expression of these molecules.

## CD40 stimulation and cellular therapies

Adoptive transfer of in vitro expanded TILs, TCR-transgenic T cells or CAR-T cells has been proposed as an approach to overcome a paucity of pre-existing T cells within the TME. While these approaches have shown consistent efficacy against hematopoietic tumors, their activity (particularly that of CAR-T cells) in solid tumors needs improvement. Given the ability of CD40-stimulating antibodies to promote DC function and the association of intratumoral DCs with T cell infiltration, it is encouraging that anti-CD40 infusion, in combination with IL-2 infusion, supports the antitumor activity of melanoma-specific TCR transgenic T cells in an IL-12- and CD80/CD86-dependent manner [71] and potentially involves CD70 [72]. Whether pretreatment with anti-CD40 can broaden the repertoire of T cells that can be expanded from patient tumor explants has not yet been studied. The aforementioned ability of CD40 to reprogram the pancreatic cancer tumor microenvironment has shown promising ability to increase the frequency and absolute number of TCR-engineered cellular products in the context of pancreatic cancer models. Anti-CD40 stimulation showed better results than anti-CSF1R on the promotion of the accumulation of transferred T cells [21]. Interestingly, IFNγ production by the engineered T cells was not enhanced with CD40 stimulation in vivo, suggesting that additional interventions will be necessary to sustain full functional activity. However, this study suggested that cellular therapies are compromised in the context of solid tumors by the ability of myeloid cells to shield tumors from T cell infiltration or limit their persistence or survival once in the tumor microenvironment. It will be important to dissect these alternatives, as it may be possible to achieve further enhancement if the results indicate that remodeling does not influence the expression of homing receptor ligands or chemokines on the tumor vasculature, for example. The activity of infused anti-CD40 in the context of CAR-T cell transfer has not been extensively addressed at this point. Rather, engineering approaches that confer the ability of CAR-T cells to express CD40L [73] or secrete agonistic CD40 antibodies [74] have shown some promise and may have the capacity to limit CD40 stimulation to the local environment of CAR-T cells, as CD40L is not expressed until CAR-T cells are activated, possibly lowering toxicity. Intriguingly, the capacity of the CD40 intracellular domains to provide costimulation of CAR-T cells has recently shown a promising ability to activate NF-κB and the subsequent expression of T cell costimulatory molecules in a manner that is discrete from CD137 (4-1BB) signaling [75] and T cells were found to be in a less differentiated state when combined with MyD88 endodomains [24].

## Delivery formats for CD40

Several studies have expanded the scope of the use of CD40 stimulation to promote antitumor immunity beyond the realm of traditional peptide/protein vaccine settings (Table 1). First, CD40 stimulation, via viral delivery of CD40L, can support the ability of viruses to promote tumor immunity in murine models and cancer patients [50, 76–80]. While not explicitly oncolytic viruses, presumably the efficacy of these approaches engages some element of antigen release by viral lytic activity and the additional innate sensing pathways activated by viral infection of cancer cells, in combination with myeloid cell activation by CD40 stimulation. Further formulations to achieve CD40 stimulation include recombinant proteins with natural trimer conformation [81], or in some instances hexamer construction [82], with the goal of more naturally and potently inducing CD40 signaling without accompanying cytokine storm toxicity that has been evident with antibody-mediated stimulation. It should be noted that some discussion is ongoing about the relative effectiveness of different CD40 cross-linking antibodies being dependent upon Fc receptor-mediated binding. On the one hand, some studies have argued that engagement of the inhibitory FcγRIIB in vivo is necessary for the functional activity of some anti-CD40 antibodies [83–86], although others have argued for FcR independence [87]. Clearly, if tumors have minimal infiltration by myeloid, NK and B cells, the opportunity to engage FcR will be limited, potentially constraining the activity of anti-CD40 to its roles in the lymph node. CD40L-based strategies, in recombinant protein form, delivered by a virus, or even as antibodies or ScFV delivered by CAR-T cells [74], may be able to circumvent this potential limitation. However, initial clinical testing of sCD40L as an agonist did not produce strong outcomes [88], prompting the development of multivalent versions of this protein. More recent studies taking advantage of aligning the antibody epitope with functional activity have suggested that improvements in antibody activity can be achieved by targeting membrane-proximal regions of CD40 [85]. Taking a different approach, CD40 oligodinucleotide aptamers have shown encouraging preclinical activity [89] with direct targeting capability to B cell lymphomas. To date, this agent has not been tested for its ability to stimulate functional activity in DCs. Notably, in the brave new world of synthetic biology, multiformat antibodies, such as bispecifics, are being used to either deliver antigenic or stimulatory payloads to CD40-expressing cells such as DCs or are being used to bring target cells close to DCs to improve their interactivity [90, 91].

**Table 1:** Developments in CD40-associated agonism

Format	Example	Ref
CD40L	Multimerization	[81, 82]
	Signaling domain in CAR-T	[24]
	Expressed by cellular therapy	[74]
	Expressed by virus	[79, 94, 97]
Agonistic anti-CD40	FcR modification	[96]
	Epitope selection	[85]
	Bispecific recombinants	[90, 91]

## Clinical outcomes and developments

The preclinical activity of anti-CD40 has made clinical investigation a high priority, especially when considering juxtaposing CD40-mediated immune “acceleration” with the success of anti-PD1 relieving immune “brakes”. Thus far, the deployment of anti-CD40 antibodies in hematological malignancies has been to either drive differentiation of the targeted B cells or induce antibody-dependent cellular cytotoxicity. For solid tumors, initial phase I testing revealed that there is a degree of toxicity for systemically delivered anti-CD40, which is not surprising given its mode of action. Nevertheless, anti-CD40 has been deployed in clinical trials either as a monotherapy where modest activity has been observed [92, 93], or more commonly, as part of a treatment regimen composed of chemotherapy [18] or with a checkpoint blockade [94]. Some significant differences have been observed in the cytokines and cellular responses between different anti-CD40 clones, which has raised speculation about the importance of the Fc domain and its affinity for FcR for the purposes of cross-linking. On the basis of these observations, second-generation engineered anti-CD40 antibodies are being tested, again generally as part of combination therapy [95, 96] with some encouraging results but also noted toxicity [95] (Table 2). There ultimately needs to be a balance between toxicity and patient outcomes, such as that seen with the enhanced activity observed with the combination of anti-PD1 and anti-CTLA4 compared to either agent alone, accompanied by increased high-grade immune-related adverse events and mortality. Further studies will need to be performed to ascertain the basis of the toxicity caused by anti-CD40 antibody therapy, as it is possible to ameliorate some elements of cytokine storms, such as blocking IL-6, which is commonly used during CAR-T therapy infusion. Alternatively, it may be preferential to deliver anti-CD40 at a lower dose via subcutaneous injection to target specific tumor deposits, draining lymph nodes, or vaccine-specific sites, which is an approach we are currently exploring in a melanoma clinical trial at our institution.

**Table 2:** Examples of active and completed trials based on varied formats of CD40 stimulation. Information from ClinicalTrials.gov

TRIAL NUMBER	AGENT	DISEASE	STATUS
NCT04491084	FLT3L; anti-CD40; stereotactic radiation	NSCLC	Recruiting
NCT01433172	GM.CD40L transfected K562 cellular vaccine with CCL21	Lung adenocarcinoma	Completed
NCT04635995	Agonistic anti-CD40+/‒ anti-PD1+/‒ anti-CD137	Advanced/metastatic malignancies	Recruiting
NCT02482168	Agonistic anti-CD40	Varied solid tumors	Completed
NCT00058799	CD40L and IL-2 transfected fibroblasts	Leukemia	Completed
NCT04364230	Agonistic anti-CD40 + peptides+polyICLC	Melanoma	Recruiting
NCT04406623	SIRPα-Fc-CD40L	Ovarian	Recruiting
NCT01561911	Agonistic Anti-CD40	Advanced malignancies	Completed
NCT01103635	Anti-CD40 + anti-CTLA4	Metastatic melanoma	Completed
NCT00020540	Flt3L and soluble CD40L	Metastatic melanoma or renal cancer	Completed
NCT03719430	Anti-CD40 and doxorubicin	Advanced sarcoma	Recruiting
NCT03329950	Anti-CD40+/‒ Ftl3L or anti-PD1 or chemotherapy	Varied malignancies	Recruiting
NCT03852511	Oncolytic adenovirus expressing anti-CD40 ab	Metastatic cancer and epithelial tumors	Recruiting

The ability to extrinsically introduce CD40L expression into the tumor by transducing with nonreplicating adenoviral infection has been tested in several small clinical trials and has shown some intriguing clinical improvements in patients with advanced malignancies [80, 94, 97]. Interrogation of local and systemic alterations that are consistent with antitumoral immunity as opposed to induced resistance mechanisms will be critical for advancing these approaches. For example, in some of the AdCD40L trials, intratumoral IL-8 and systemic IL-8 were observed to be modulated in some patients [80, 98]. IL-8 is a well-described chemoattractant for neutrophils/granulocytic MDSCs that can have both tumor- and immune-constraining influences.

## Further considerations in the development of CD40 stimulation

Aside from the toxicity observed with targeting CD40 stimulation for immune-oncology purposes, our knowledge of the immunobiology of CD40 stimulation should provide some opportunities for optimization in the clinical setting. One of the most important considerations is the sequencing of CD40 stimulation, which will be context dependent when delivered as part of either conventional therapy or vaccination. Given that CD40 stimulation rapidly promotes the maturation of DCs, which initiates their migration to lymph nodes and increases the expression of costimulatory molecules and cytokines and that mature DCs have a reduced capacity to acquire antigens, providing CD40 stimulation prior to or coincident with conventional therapies may not be optimal. However, if CD40 stimulation is used to remodel the tumor microenvironment to allow more chemotherapy access, then sequencing CD40 prior to chemotherapy may be logical. However, increased toxicity has been reported when CD40 stimulation is introduced prior to chemotherapy [99]. Similar to cancer vaccines, the format of the antigen will be an important consideration for the timing of CD40 stimulation. Preprocessed MHC class I- or MHC class II-restricted peptides will be less dependent upon engulfment by DCs or macrophages, while recombinant proteins or tumor lysates will likely be best deployed in the context of relatively immature DCs that are subsequently activated via CD40 stimulation. Clearly, clinical trials will be needed to determine the relative efficacy with an empirical approach. It should also be noted that CD40 stimulation has been shown to have some antagonistic activity on T cells in animal models. In one report, infusion of agonistic CD40 as a monotherapy in tumor-bearing mice resulted in functional deletion of tumor-specific CD8 + T cells [100]. This situation was alleviated by vaccination with a recombinant virus expressing the targeted tumor antigen, suggesting that either virally induced inflammation or providing additional antigen was needed, as seen with the benefits of cotargeting PRR with CD40 stimulation. In unpublished studies from our lab intended to understand how long CD70 can be induced on DCs, it was observed that prevaccination infusion of anti-CD40 instilled a period of tolerance lasting 2–3 weeks that limited T cell expansion to peptide/protein vaccination or T cell responses to tumors (*McClintic, H, Francica, B and Bullock, TNJ; manuscript in preparation*). Thus, while these studies were performed in mice with transplantable tumors, they suggest that the sequencing of CD40 stimulation may be very important with respect to its impact on T cell responses.

## Concluding remarks

Considerable attention has been given to the beneficial effects of removing the inherent “brakes” on antitumor immunity, either in the context of promoting preexisting T cell responses or supporting vaccination approaches. CD40 stimulation provides an opportunity to concurrently modulate the state of primarily myeloid cells within the TME, allowing for increased T cell priming, infiltration and functional activity. Advances in our knowledge of CD40 biology combined with results and immune correlate material from early-stage clinical trials will inevitably provide opportunities to promote the outcomes of CD40 stimulation whether in combination with traditional cancer vaccines or with interventions that result in autovaccination. Considerable opportunities exist for further development of rationally designed bispecifics or conjugates and incorporation into cellular therapies. This knowledge will likely result in the improved design, execution and outcomes of second-generation clinical trials and the development of novel treatment modalities that will improve clinical outcomes in patients.
